# Insurance Sanatorium Construction: Hints to Architects by a Sanatorium Superintendent

**Published:** 1913-10-04

**Authors:** 


### INSURANCE SANATORIUM CONSTRUCTION.

### Planning Hints to Sanatorium Architects.

By A SANATORIUM SUPERINTENDENT.

This gaudeficent summer it has been the not unpleasant task of sundry municipal and other architects to travel about the country looking at sanatoriums with a view to obtaining or testing impressions as to what is desirable in the way of design; these impressions destined, of course, to be embodied in an eclectic plan of construction. Now, electicism is admittedly a good thing, and so, it may be supposed, one can have too much of it. But in the case under notice, that of the erecting of sanatoriums to meet the provisions and requirements of the National Insurance Act, there are plenty of safeguards against such excess. Peculiar local conditions are the one that suggests itself first. A small site, or the obligation to make use of some existing structure, or the high degree of availability of some particular building material, may put out of court various recommendations. By now, too, the general design of a fifty to a hundred bed sanatorium is fixed as regards the main points.

### The One Roof Plan.

Separate huts or châlets are pleasant for the patient and excellent in the matter of ventilation, but they make attendance a difficult thing and proper discipline very uncertain.* All the patients, therefore, must be under one roof, and this is preferably the case even where advanced cases are treated separately in the same institution. There must be a thought-out system of strict separation of the sexes, although the use of a common recreation room, say, for one hour in the day, if the room is readily observable, presents no objection, and is in all probability an advantage from the particular point of view. More effectual than any other restraint to the exuberance of architectural fancy, however, is that of expense. It can never be repeated often enough that the tuberculosis problem, which the "Sanatorium Benefit" Section of the Act is designed to solve, is even more an economic than a medical one. That is, if the nation is going really to provide for its consumptives and its scrofulous and crippled children, it must see to it that not even a sixpence is spent unless strictly required. Gifts of elaborate buildings may prove to be Greek gifts, since money cannot be permanently forthcoming for their upkeep.

The last circular of the Local Government Board is a useful reminder in this connection, although that distinctly flatters the possibilities. Every subsoil is not capable of affording a firm foundation by dint of simple superimposing of a layer of concrete. To the objection that careful search will reveal the natural formation required in every instance, the reply is that the selection of sanatorium sites is hampered practically in many ways. Local residents object; water and sewage mains must be available if the £150 a bed estimate is to be approached; in this climate southern aspects are imperative, and so on. What will be really helpful, however, is to indicate means by which expense may be kept down, means which are also practicable, and likewise very generally overlooked by responsible people.

### A Window Paradox.

In the first place, paradoxical as it may seem, specially constructed sanatoriums are very generally fitted with too many windows. Look at these buildings at any time except the noontide of a midsummer day, and you will find most of the smaller windows shut. Inspect many of the older established private sanatoria and you will find that they are converted private houses, with not more than one window per room, or two per short passage. Yet they can show a record of results quite up to the average. It is probably safe to say that from the number of windows shown in any architect's design a proportion of one-quarter to one-third may be deducted with no harm at all; and, therefore, with great good, because glass costs far more to begin with than brick or corrugated iron, and similarly to keep up (by weekly cleaning) and in repairs. In spite of all patent fasteners, windows blow off or blow in very often in sanatoriums, and the joiner's work necessary to replace or reglaze them cannot be got under from 7½d. to 9d. an hour.

### The Balcony Fad.

The next point over which the amateur in sanatorium management goes astray is the provision of balconies. He thinks these an essential, because, he will say, "You can pull the beds out on to them." Really, the motto—"Let him have air, he'll straight be well," was a most mischievous one to choose. Because, as some of the then air enthusiasts are now coming to see, the question of rest and exercise is an essential too, and the external air has no advantage over the atmosphere of a suitably prepared room with good cross-ventilation. On the other hand, patients in beds pulled out on to a balcony sit up, talk and shout to each other, even throw things, unless someone is constantly on the watch. Now, the reason for confining a consumptive to bed is to reduce his temperature; the quieter he is kept the better. "Typhoid rest," as practised at Frimley, may not be necessary, but short of this the stiller the patient is the sooner he will be up. A row of white beds outside a long building may have a good effect spectacularly, but in no other way. In competent hands it is only here and there a case that is treated in this way—for instance, a very chronic one under treatment for many months, such as is not likely to be met with in public sanatoria. Economically, again, frequent bodily shifting of beds makes work, and wears out floors and castors.

### Ward Kitchens and Duty Rooms.

A point in which our good friends the architects are liable to complicate construction unnecessarily is the provision of ward kitchens and "duty rooms." Nearly all of these become, in an extravagantly managed sanatorium, a place where a nurse burns coal to waste; and in an economically run one, a receptacle for housemaids' brooms and other impedimenta. The proportion of sanatorium (not advanced) cases on special diets is exceedingly small, and all diets are much better served from the kitchen. One duty room is a necessity when there are a hundred beds for the use of the night nurse who is then required. But more is space wasted.

Conversely designers are apt to stint, or even to forget, drying accommodation for the patients' clothes. In this climate, a big drying room to hang up overcoats, etc., in, is a sanatorium requisite. And plenty of fixed wash-hand basins are a boon, especially now that graduated labour makes so many dirty hands every day.

*De minimis non curat lex*, it is said; but that is where scientific professions differ from non-scientific ones. If certain small points are seen to, very simple buildings will suffice, thus leaving all the more money for grounds and land. Plenty of grounds and land mean facilities for employment of ex-patients and "after-care" generally. And "after-care," as every tuberculosis officer is coming in his heart to acknowledge, is the real crux of the matter as regards the saveable consumptive. But we are getting now on to another story.

*[Readers of our article on "Queen Mary's Hospital for Children, Carshalton," which appeared in The Hospital of July 26, will remember that the cottage-block system which prevails there was said to cause no serious administrative difficulty. But, of course, conditions which suit a hospital for children do not necessarily apply to a sanatorium for adult consumptives.—Ed. The Hospital.]

